# Hepatocyte-derived lipid droplets in MASH: macrophage sensing and inflammation

**DOI:** 10.3389/fimmu.2026.2011384

**Published:** 2026-10-26

**Authors:** Di Jin, Yinan Zhao

**Affiliations:** 1 Teaching and Research Division of Internal Medicine, Hubei College of Chinese Medicine, Jingzhou, China; 2 Clinical Medical College of Qinghai University, Xining, China

**Keywords:** immunometabolism, lipid droplets, macrophages, MASH, MS4A7, NLRP3 inflammasome, TREM2

## Abstract

Metabolic dysfunction-associated steatohepatitis (MASH) develops through interactions between hepatocyte injury and hepatic immune responses. Lipid droplets (LDs) exposed or released from damaged hepatocytes provide a potential link between lipid accumulation and macrophage-mediated inflammation. Experimental studies indicate that these structures can both promote disease-associated macrophage responses and attenuate inflammatory signaling. This mini-review examines how hepatocyte-derived LDs influence macrophage sensing, uptake, and inflammatory responses in MASH. We discuss the contribution of membrane-spanning 4-domains A7 (MS4A7) to NLRP3 inflammasome activation and disease progression, alongside evidence that LD treatment suppresses inflammatory responses in cultured macrophages. Triggering receptor expressed on myeloid cells 2 (TREM2) supports this suppression without being required for LD uptake in the tested system, while reduced MS4A7 expression contributes to the attenuation of inflammatory signaling. These findings distinguish LD internalization from its downstream immune effects and warrant separate consideration from the efferocytosis of intact apoptotic hepatocytes. We examine experimental conditions and macrophage states as possible explanations for differing outcomes and identify unresolved questions about LD recognition and relevance to human disease. Finally, we consider therapeutic strategies that limit pathogenic macrophage activation while preserving beneficial clearance functions. Defining the molecular basis of LD sensing and establishing its consequences *in vivo* will be essential for translating this form of hepatocyte–macrophage communication into immunological interventions for MASH.

## 1 Introduction

Metabolic dysfunction-associated steatotic liver disease (MASLD), formerly termed nonalcoholic fatty liver disease (NAFLD), is a common chronic liver disease (1). A meta-analysis of 92 studies using the NAFLD definition estimated a global prevalence of 30.05%, increasing from 25.26% in 1990–2006 to 38.00% in 2016–2019 (2). Its pathological spectrum extends from steatosis to hepatocyte ballooning and lobular inflammation, which characterize metabolic dysfunction-associated steatohepatitis (MASH) (1, 3). Persistent injury may promote fibrosis and ultimately cirrhosis, highlighting the importance of immune mechanisms in disease progression (4, 5).

Lipid droplets (LDs) store neutral lipids within hepatocytes, but their significance changes when injury exposes them to surrounding immune cells (6). Experimental evidence identifies extracellular LDs as signals that recruit monocytes and promote macrophage responses associated with steatohepatitis (7). This shifts attention from the amount of fat retained within hepatocytes to how lipid material is encountered and processed by macrophages (8). Yet the consequences of this encounter are not uniformly proinflammatory (8). In cultured macrophages, LD exposure can suppress inflammatory signaling, indicating that the response to lipid cargo cannot be inferred from its uptake alone (9).

These observations raise questions about the relationship between lipid recognition, internalization, and immune activation. LD uptake must also be distinguished from efferocytosis of intact apoptotic hepatocytes, a process that can protect against steatohepatitis in experimental models (10). This mini-review examines the origin of hepatocyte-derived LDs, macrophage sensing and uptake, and the evidence for opposing inflammatory responses. It also considers experimental limitations and therapeutic opportunities, with particular emphasis on preserving clearance functions while restricting inflammation that sustains MASH.

## 2 Hepatocyte-derived lipid droplets as immune signals

Within hepatocytes, LDs consist of a neutral lipid core rich in triglycerides and cholesteryl esters, enclosed by a phospholipid monolayer and associated proteins (9). Esterification into triglycerides can buffer excess fatty acids, so LD accumulation does not necessarily indicate that the stored lipid is itself inflammatory (11). In obese mice fed a methionine- and choline-deficient diet, suppression of triglyceride synthesis reduced steatosis but worsened liver injury and fibrosis (12). The immunological significance of LDs therefore depends partly on whether they remain contained within viable hepatocytes or become accessible after cellular injury (11, 13).

Histology provides evidence for this change in accessibility. Hepatic crown-like structures contain macrophages arranged around damaged hepatocytes with large LDs and have been described in both experimental steatohepatitis and human liver samples (14). Such structures place macrophages in direct contact with lipid residues and other cellular components of dying cells. Cholesterol crystals within hepatocyte LDs further distinguish some inflammatory lesions from simple steatosis (15). A recent study linked increased LD cholesterol to MASH and fibrosis in human samples, supporting the relevance of subcellular lipid distribution to disease severity (16). These observations implicate lipid composition and physical state, alongside lipid quantity, in the local immune response.

Intervention studies strengthen the case for LDs as biologically active signals. Administration of LDs isolated from steatotic mouse livers promoted monocyte recruitment and the accumulation of macrophages expressing triggering receptor expressed on myeloid cells 2 (TREM2), while aggravating liver injury in a MASH model (7). However, isolated LDs also altered transcription and reduced inflammatory responsiveness in cultured macrophages (9). These findings establish biological activity without identifying which components of the LD preparation initiate the response.

LDs should also be distinguished from intact apoptotic hepatocytes and extracellular vesicles. The latter can carry inflammatory signals from lipotoxic hepatocytes through a separate route (17), whereas engulfment of whole apoptotic cells combines lipid uptake with recognition of cell death signals (10). For isolated LD preparations, the relative contributions of neutral lipids, surface phospholipids, associated proteins, and co-isolated cellular material remain unresolved. Establishing which components are sensed, and whether experimental doses and exposure patterns reflect those in diseased liver, is essential before assigning LDs a defined receptor-mediated inflammatory function.

Human studies provide additional evidence of macrophage remodeling at this interface. In liver biopsies from seven patients with MASH and seven non-MASH controls, Zhou et al. found elevated MS4A7 expression and increased abundance of MS4A7-positive cells; within the MASH group, MS4A7 expression correlated with TREM2 and COL1A1 (7). Chan et al. identified a macrophage subset expressing TREM2, CD9, and glycoprotein NMB (GPNMB) in human fatty liver and found that hepatic CD14-positive monocyte/macrophage abundance correlated with steatosis severity in a cohort with generally minimal fibrosis (18). However, in a separate bariatric-surgery cohort of ten patients with NASH, Fred et al. detected TREM2-positive/CD9-positive cells in only approximately 1% of recovered macrophages (19). These cohort-specific findings caution against assuming that TREM2-associated macrophages expand uniformly across human disease or reproduce the populations observed in mouse models.

Human tissue studies also offer functional evidence for macrophage responses to lipid loading. In an analysis of 170 NAFLD liver biopsies, Govaere et al. associated macrophage scavenger receptor 1 (MSR1) expression with foamy macrophages and the severity of steatosis and steatohepatitis. Antibody-mediated MSR1 blockade reduced foamy macrophage formation and inflammation in lipid-loaded human liver slices (20). More recently, Boesch et al. combined single-nucleus and spatial profiling to identify GPNMB-positive macrophages within lipogranulomas and regions containing cellular debris. Lipid loading of human precision-cut liver slices promoted macrophage aggregation and early lipogranuloma formation (21). These findings support macrophage responses to lipid-rich environments in human tissue, although the slice experiments examined lipid loading rather than exposure to purified, intact hepatocyte-derived LDs.

Collectively, human findings support the spatial association of macrophages with lipid-rich lesions and disease-related changes in their lipid-handling programs. These studies do not establish that uptake of intact hepatocyte-derived LDs activates MS4A7–NLRP3 signaling or induces TREM2-dependent inflammatory attenuation in human liver macrophages. Establishing these responses will require direct uptake and functional assays using compositionally characterized human hepatocyte LDs and defined hepatic macrophage populations, with particular attention to differences between Kupffer cells and recruited macrophages.

## 3 Macrophage sensing and uptake of lipid droplets

Macrophages encountering hepatocyte LDs differ in developmental origin and location within the injured liver (22). In experimental fatty liver, resident Kupffer cells are progressively replaced by recruited macrophages, some acquiring Kupffer cell features and others adopting distinct lipid-associated programs (23, 24). Remmerie et al. identified an osteopontin-expressing population associated with regions of increased desmin staining, linking macrophage heterogeneity to the fibrotic microenvironment (25). Consequently, LD exposure should be interpreted alongside macrophage identity: enrichment of a lipid-associated transcriptional signature does not establish which receptor mediates uptake or whether the resulting response is injurious.

TREM2 is a plausible lipid sensor, although much of the direct ligand evidence comes from other tissues. In reporter assays accompanying studies of experimental neurodegeneration, TREM2 responded to anionic and zwitterionic lipids, whereas the R47H variant impaired lipid recognition (26). TREM2 signals through DNAX-activating protein of 12 kDa (DAP12), which recruits spleen tyrosine kinase and transmits ligand recognition into intracellular responses (26). These findings provide a molecular rationale for investigating TREM2 at the hepatocyte–macrophage interface. They do not, however, establish direct recognition of intact hepatocyte LDs. Whether particular phospholipids or surface proteins enable such recognition remains unresolved, as does the possibility of compensation by other uptake receptors.

Direct uptake experiments help separate recognition from internalization. Using an inverted culture system to bring buoyant liver-derived LDs into contact with adherent mouse bone marrow-derived macrophages, Zhou et al. observed rapid engulfment within three hours. Deletion of either *Trem2* or *Ms4a7* did not appreciably impair LD uptake. Nevertheless, TREM2 deficiency weakened the suppression of inflammatory signaling following LD exposure (9). Thus, preserved uptake can coexist with altered immune responses. Membrane-spanning 4-domains subfamily A member 7 (MS4A7) also interacts with NLR family pyrin domain containing 3 (NLRP3) and facilitates inflammasome activation, supporting a role in intracellular inflammatory signaling without establishing it as an extracellular LD receptor (7).

Candidate surface uptake pathways include the scavenger receptors MSR1 and CD36. MSR1 blockade limits macrophage lipid accumulation and inflammation in NAFLD-related experimental systems and human liver slices (20), providing disease-relevant support for investigating its role in LD handling. However, intracellular LD accumulation after lipid loading is not equivalent to ingestion of extracellular hepatocyte LDs. CD36 recognizes defined oxidized phospholipids that mediate macrophage binding to oxidized lipoproteins (27), making it a candidate if corresponding ligands are accessible on LD surfaces. This extrapolation requires demonstration of ligand availability, receptor–LD binding, and receptor-dependent internalization; neither receptor should be assigned a proven role in intact hepatocyte LD uptake on the basis of these studies alone.

This distinction explains why isolated LD uptake cannot substitute for assays of whole-cell efferocytosis. Wang et al. showed that hepatocyte-derived sphingosine-1-phosphate increased macrophage TREM2 expression and that TREM2 supported clearance of lipid-laden apoptotic hepatocytes. With prolonged hypernutrition, TNF and IL-1β promoted ADAM17-dependent TREM2 shedding, impairing clearance and sustaining inflammation (10). These experiments assessed recognition and disposal of dying cells carrying multiple membrane and intracellular signals. Their dependence on TREM2 therefore does not contradict preserved uptake of isolated LDs in a different experimental system. Cargo identity is central to interpreting the apparent discrepancy.

Established apoptotic-cell recognition pathways provide a separate comparator. T-cell immunoglobulin and mucin-domain-containing protein 4 (TIM4) binds exposed phosphatidylserine (28), while MER receptor tyrosine kinase (MerTK) supports engulfment through phosphatidylserine-binding bridging proteins such as GAS6 and protein S. Cooperation between TIM4-mediated tethering and MerTK-mediated engulfment was demonstrated in mouse resident peritoneal macrophages (29). These findings concern apoptotic membranes rather than hepatic LDs. Their relevance to isolated LD uptake would require evidence that phosphatidylserine is accessible on the LD monolayer or associated membranes and that the necessary bridging ligands are available. An LD phospholipid monolayer should not be assumed to reproduce the recognition surface of an apoptotic cell.

Internalization also precedes a separate metabolic step. In cultured macrophages, lysosomal acid lipase supported degradation of engulfed LDs, and its inhibition reduced LD hydrolysis and fatty acid release. However, blocking lysosomal hydrolysis did not abolish the attenuation of NLRP3 inflammasome activation caused by LD exposure (9). Lipid digestion and immune regulation can therefore be experimentally dissociated. Future studies should quantify surface binding, internalization, lysosomal processing, and cytokine responses separately in defined hepatic macrophage populations. Candidate receptor blockade or deletion should be paired with rescue experiments and measurements that distinguish surface adhesion from internalization. Cargo tracing should also establish that the transferred material remains an intact LD, rather than reflecting lipid exchange or uptake of co-isolated debris. Tracking hepatocyte-derived cargo *in vivo* would further distinguish transferred LDs from droplets synthesized within macrophages after lipid uptake. This separation of cellular entry, processing, and signaling provides the framework for interpreting the divergent inflammatory outcomes summarized in **Figure 1**.

**Figure 1:**
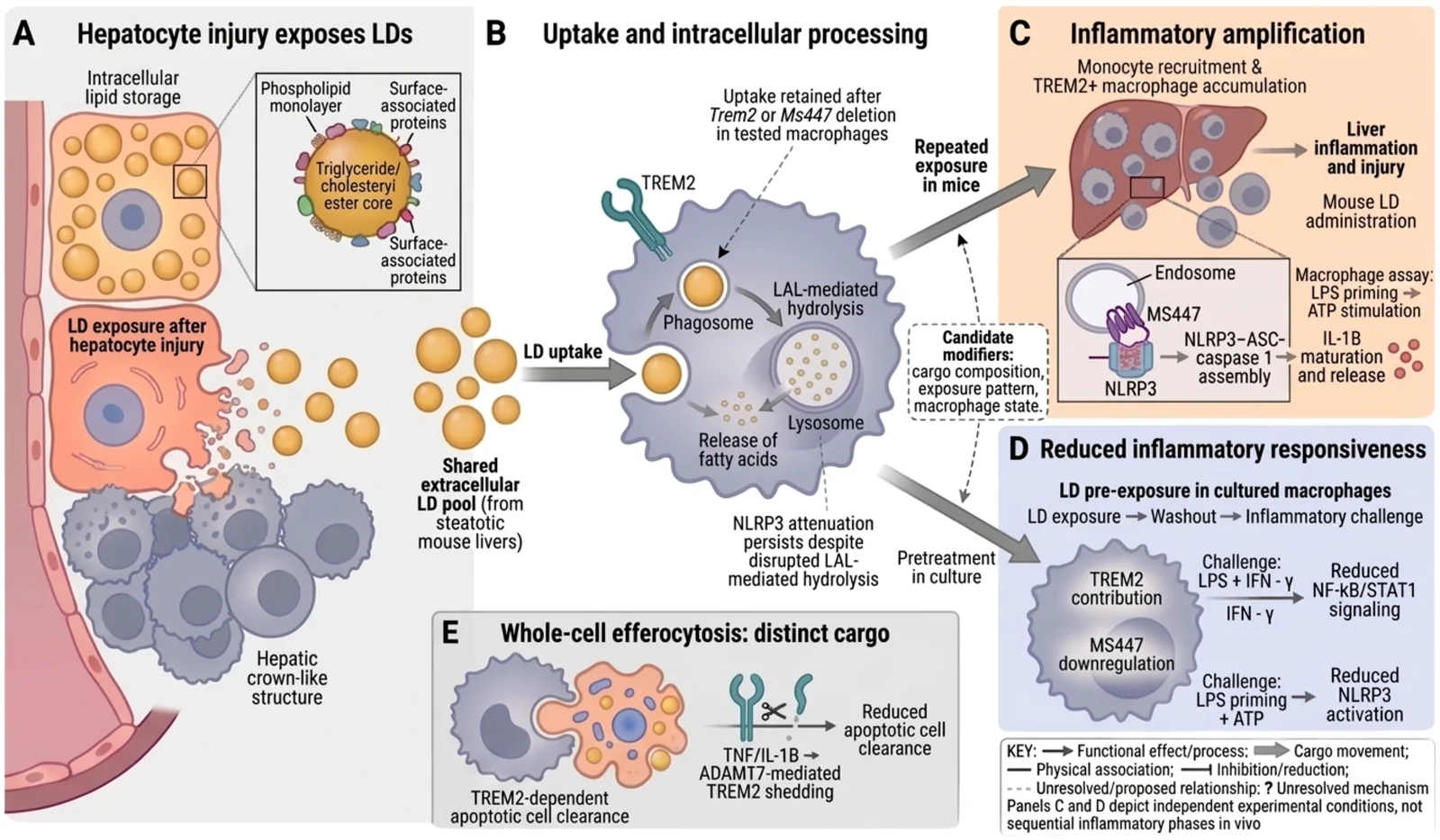
Hepatocyte-derived lipid droplets and macrophage inflammatory responses in MASH. **(A)** Hepatocyte injury exposes lipid droplets (LDs), with macrophages accumulating in hepatic crown-like structures. **(B)** Macrophages internalize LDs isolated from steatotic mouse livers and process their lipid cargo through lysosomal acid lipase (LAL). Uptake is retained after Trem2 or Ms4a7 deletion, whereas attenuation of NLRP3 activation can persist despite disrupted LAL-mediated hydrolysis. **(C)** Repeated LD administration in mice promotes monocyte recruitment, TREM2^+^ macrophage accumulation, and liver inflammation. Complementary macrophage assays identify endosomal MS4A7–NLRP3 interactions that facilitate inflammasome assembly and IL-1β maturation and release. **(D)** LD pre-exposure attenuates NF-κB/STAT1 signaling and NLRP3 activation following inflammatory challenge, with contributions from TREM2 and MS4A7 downregulation. **(E)** TREM2 supports clearance of lipid-laden apoptotic hepatocytes, whereas TNF/IL-1β-induced, ADAM17-mediated TREM2 shedding impairs this process. Panels C and D depict independent experimental conditions: repeated LD administration in mice **(C)** and LD pretreatment followed by inflammatory challenge in cultured macrophages **(D)**. They do not represent sequential inflammatory phases *in vivo*. Cargo composition, exposure pattern, and macrophage state remain proposed modifiers of these responses. Created by BioRender (Agreement number: HB2ABKBYAM).

## 4 Lipid droplets and macrophage inflammation

LD uptake can reshape macrophage inflammatory responses, but the direction of this response cannot be inferred from lipid accumulation alone. Studies linking hepatic LDs to macrophage recruitment and MS4A7-dependent inflammasome activation coexist with evidence that LD pretreatment suppresses subsequent inflammatory signaling (7, 9). These findings highlight the need to distinguish mechanisms of inflammatory amplification from those of attenuation, while considering how experimental design influences the observed outcome.

### 4.1 MS4A7–NLRP3 inflammasome activation

For NLRP3, transcriptional priming and inflammasome activation are distinct regulatory steps: signals mediated by nuclear factor κB (NF-κB) increase the availability of NLRP3 and pro-IL-1β, whereas a subsequent stimulus such as ATP enables inflammasome activation (30). This distinction matters when interpreting LD experiments, because increased inflammatory transcripts, caspase-1 cleavage, and cytokine release report different stages of the response.

Zhou et al. identified MS4A7 as a pathogenic component of the macrophage program induced during experimental MASH (7). Global or myeloid Ms4a7 deletion reduced liver inflammation and fibrosis (7). In macrophages challenged with lipopolysaccharide (LPS) and ATP, deficiency diminished caspase-1 and IL-1β cleavage despite relatively modest changes in inflammasome component expression. MS4A7 localized to endosomes and associated with endogenous NLRP3; ATP strengthened this association (7, 31). These observations support a role in organizing inflammasome activation at intracellular membranes. Moreover, MCC950 improved MASH pathology in wild-type mice but provided no additional protection in Ms4a7-deficient animals, consistent with substantial overlap between their pathogenic effects (7, 31).

The evidence therefore connects MS4A7 to inflammatory execution more directly than to LD recognition (7, 9). Physical association with NLRP3 does not identify the LD constituent responsible for initiating this response (7, 9). Likewise, the absence of additional benefit from combined interventions supports pathway convergence but cannot establish that every action of MS4A7 requires NLRP3 (9). Measurements of receptor engagement, membrane recruitment, and inflammasome assembly remain necessary to connect extracellular cargo to this intracellular machinery (7, 9, 32). TREM2-positive macrophage accumulation should also be distinguished from activation of a specific inflammatory pathway within those cells (33).

### 4.2 Suppression of inflammatory signaling following lipid droplet exposure

A different response emerged when macrophages encountered LDs before inflammatory stimulation. In the subsequent study by Zhou et al., bone marrow-derived macrophages received liver LDs for three hours, followed by washout and challenge (9). Pretreatment reduced NF-κB and signal transducer and activator of transcription 1 (STAT1) signaling after LPS plus interferon-γ (IFN-γ) and diminished IL-1β processing and release after LPS priming followed by ATP. Suppression also occurred with TNF plus IFN-γ, arguing against simple sequestration of LPS by the droplets. LD exposure lowered MS4A7 expression, while TREM2 deficiency weakened inflammatory attenuation despite preserved uptake. LD exposure also reduced MS4A7–NLRP3 association and provided no additional inflammasome suppression in MS4A7-deficient macrophages. Disrupting lysosomal acid lipase (LAL)-mediated hydrolysis did not abolish NLRP3 suppression (9).

Conditioned-medium experiments also showed reduced hepatocyte toxicity (9). Nevertheless, lower cytokine output and reduced toxicity in culture do not demonstrate resolution of established MASH. Whether the response preserves antimicrobial functions or alters communication with stellate cells remains unknown.

Nor should this phenotype automatically be called endotoxin tolerance. Classical tolerance involves selective regulation of inflammatory and antimicrobial genes through stimulus-induced chromatin changes (34). Establishing an analogous process after LD exposure would require measurements of response persistence, chromatin accessibility, and responses to repeated or unrelated challenges. Such experiments would distinguish temporary attenuation from a durable change in macrophage inflammatory capacity.

### 4.3 Interpreting differences between experimental findings

The apparent discrepancy partly reflects what each experiment measures. Repeated LD administration interrogates recruitment and injury across an intact liver, whereas pretreatment assays measure how individual macrophages respond to a later stimulus (7, 9). Increased macrophage abundance and lower cytokine production per cell could therefore coexist. This possibility does not establish a temporal switch from inflammatory amplification to resolution, and neither study directly tests that sequence.

Exposure order and delivery introduce additional differences. The *in vivo* injury experiment used four daily LD injections in mice with diet-induced MASH (7), whereas cultured macrophages received LDs for three hours before washout and subsequent inflammatory challenge (9). Prior uptake may therefore alter responsiveness to a later stimulus without predicting the consequences of repeated exposure during ongoing tissue injury. Tail-vein administration and inverted culture also produce different patterns of particle–cell contact, with systemic distribution and clearance operating only in the former setting. Particle number, lipid mass, size distribution, and internalized cargo per viable cell require calibration before a dose- or exposure-dependent switch can be inferred (7, 9).

Cargo composition is another plausible source of variation. Sakuma et al. linked hepatic LD cholesterol accumulation to MASH development and showed that interventions lowering this pool reduced disease features (16). These findings justify measuring cholesterol and phospholipid composition alongside triglyceride content, but do not establish cholesterol as the cause of the opposing macrophage responses (35, 36). Oxidized lipids, associated proteins, and residual cellular material likewise remain candidates requiring controlled comparisons (9, 37). Because experimental preparations originate from whole steatotic livers, the contribution of individual donor cell populations also requires clarification.

Recipient identity deserves equal attention. Recruited hepatic macrophages include populations distinct from resident Kupffer cells, with different transcriptional programs and tissue distributions (25). Responses obtained in bone marrow-derived macrophages cannot therefore be assigned uncritically to every hepatic macrophage subset. Comparisons should account for developmental origin, prior inflammatory exposure, and local interactions with hepatocytes and stellate cells. Importantly, NLRP3 suppression was also reproduced in primary liver macrophages isolated from MASH mice (9); BMDM origin alone therefore cannot account for the discrepancy. These assays did not resolve responses across individual hepatic macrophage subsets, and isolation removes the ongoing recruitment and multicellular feedback present *in vivo*.

Comparisons should expose defined macrophage populations to the same characterized LD preparation before, during, and after inflammatory stimulation. Particle abundance, lipid mass, endotoxin contamination, and viability require independent assessment, alongside mock preparations to detect isolation-related artifacts. The *in vitro* study assessed LD fraction purity using subcellular markers, and suppression after TNF plus IFN-γ stimulation argues against LPS sequestration as a sufficient explanation (9). Nevertheless, viability-normalized cytokine measurements and quantitative assessment of residual inflammatory material are needed to evaluate preparation- or assay-related effects; contamination and cytotoxicity are not established explanations for the divergent findings. Combining cytokine assays, cargo tracking, and MS4A7 perturbation could separate altered delivery from altered signaling. Testing these mechanisms in human liver macrophages would help establish their relevance to MASH. **Table 1** summarizes the experimental distinctions and their limitations.

**Table 1:** Experimental evidence on lipid droplet uptake, efferocytosis, and macrophage inflammation in MASH.

Experimental setting	Principal findings	Interpretation and limitations	References
Diet-induced mouse MASH; repeated administration of LDs isolated from steatotic livers; macrophage assays.	LD administration increased hepatic TREM2+ macrophage accumulation and liver injury. MS4A7 promoted NLRP3 activation; global or myeloid *Ms4a7* deletion reduced inflammation and fibrosis.	Links LD exposure to liver inflammation, but does not establish MS4A7 as a receptor mediating LD uptake.	(7)
Murine macrophages; 3-h LD pretreatment in BMDMs, followed by washout and inflammatory challenge.	LD pretreatment reduced NF-κB/STAT1 signaling and NLRP3 activation. TREM2 contributed to suppression but was dispensable for uptake. MS4A7 downregulation contributed to attenuation; blocking LAL-mediated hydrolysis did not abolish NLRP3 suppression. NLRP3 suppression was also observed in primary liver macrophages isolated from MASH mice.	Demonstrates reduced responsiveness to subsequent stimulation in culture; does not establish resolution of MASH *in vivo*.	(9)
Diet-induced mouse steatohepatitis; efferocytosis assays using lipid-laden apoptotic hepatocytes.	TREM2 supported apoptotic hepatocyte clearance. TNF and IL-1β promoted ADAM17-dependent TREM2 shedding, impairing efferocytosis and sustaining liver inflammation.	Examines whole apoptotic hepatocytes, rather than isolated LDs; TREM2 dependence cannot be directly extrapolated to LD uptake.	(10)
Hepatic LD analysis and interventions reducing LD cholesterol in experimental MASH.	Reducing hepatic LD cholesterol through coenzyme A synthase knockdown or bempedoic acid treatment limited MASH and fibrosis.	Supports the importance of LD composition, but does not establish cholesterol as the explanation for divergent macrophage responses to isolated LDs.	(16)

Differences in exposure sequence, delivery, effective dose, cargo composition, macrophage state, and assay controls are discussed in Section 4.3. These factors should be considered potential explanations rather than established causes of the divergent outcomes.

ADAM17, a disintegrin and metalloproteinase 17; BMDM, bone marrow-derived macrophage; IL-1β, interleukin-1β; LAL, lysosomal acid lipase; LD, lipid droplet; MASH, metabolic dysfunction-associated steatohepatitis; MS4A7, membrane-spanning 4-domains A7; NF-κB, nuclear factor κB; NLRP3, NLR family pyrin domain-containing 3; STAT1, signal transducer and activator of transcription 1; TNF, tumor necrosis factor; TREM2, triggering receptor expressed on myeloid cells 2.

## 5 Therapeutic implications

Therapeutic targeting of the LD–macrophage interface should preserve lipid disposal while limiting inflammatory injury. In mice, coenzyme A synthase knockdown and bempedoic acid reduced hepatic LD cholesterol and prevented steatohepatitis and fibrotic responses (16). These findings favor investigating the composition of retained lipid cargo alongside its abundance (38, 39). They also suggest that therapeutic effects on LD cholesterol deserve direct measurement, because changes in total hepatic lipid content may conceal redistribution between intracellular compartments (38, 39). Whether lowering LD cholesterol reverses established fibrosis in patients requires clinical investigation (40).

The MS4A7–NLRP3 pathway offers a means of intervening after cargo uptake. Genetic loss of MS4A7 reduced experimental MASH, and NLRP3 inhibition provided no additional protection in deficient mice (7). This convergence supports comparing MS4A7-directed approaches with inflammasome inhibition, with attention to liver delivery, target engagement, and preservation of macrophage clearance functions. Because isolated LD uptake persisted after *Ms4a7* deletion (9), suppression of inflammatory signaling may be achievable without preventing ingestion; whether this separation persists in diseased human macrophages remains uncertain.

Preserving TREM2-dependent efferocytosis provides a complementary strategy. Lipid nanoparticles delivering cleavage-resistant TREM2 mRNA enhanced apoptotic cell clearance and reduced inflammation in mouse MASH and atherosclerosis models (41). These findings provide preclinical support for receptor stabilization, but concern apoptotic-cell clearance rather than isolated LD uptake. Durability and off-target effects require evaluation during prolonged treatment in metabolic disease. Native LD administration is not an equivalent therapeutic approach: LD pretreatment attenuated inflammatory signaling in culture, whereas repeated administration aggravated liver injury in mice (7, 9). Identifying the components responsible for attenuation would be preferable to administering heterogeneous liver-derived preparations (42).

Clinical development must establish benefits beyond changes in cytokine output. Resmetirom and semaglutide improved liver histology in randomized trials (43, 44), although those results do not demonstrate mediation through LD–macrophage signaling. Future studies could test whether selective immune intervention adds benefit to metabolic therapy, combining histological endpoints with measurements of LD composition, inflammasome activity, and efferocytic capacity (45, 46). Prospective analyses should distinguish reduced macrophage recruitment from altered activity within cells (46). Such measurements would require validation before use for patient selection or response monitoring. Treatment should ultimately reduce hepatocyte injury and fibrosis while retaining effective debris clearance and antimicrobial defense (47, 48).

## 6 Discussion

LDs link hepatocyte injury to macrophage responses through mechanisms that extend beyond intracellular lipid storage. Experimental studies associate extracellular LD exposure with macrophage accumulation and liver injury, yet also show that prior LD uptake can attenuate subsequent inflammatory signaling. These findings concern different biological scales and experimental schedules; they do not establish successive inflammatory and resolving phases. The distinction between cargo ingestion and signaling is equally important. TREM2 and MS4A7 influence inflammatory responses despite being dispensable for isolated LD uptake in tested macrophages. Accordingly, the therapeutic value of targeting either protein cannot be inferred from macrophage abundance or lipid loading alone.

Further progress requires establishing which features of native hepatocyte LDs determine their immunological effects. Tracing hepatocyte-derived cargo *in vivo* and comparing defined LD preparations across human macrophage populations would help resolve questions of cellular origin, composition, and recipient identity. We predict that macrophage-selective MS4A7 inhibition reduces inflammatory injury while preserving LD disposal and TREM2-dependent clearance of apoptotic hepatocytes. This requires simultaneous assessment of uptake, cytokine production, hepatocyte viability, and tissue repair. Longitudinal studies should determine whether changes in macrophage signaling precede measurable fibrosis regression and sustained recovery. Benefits of engineered TREM2 in separate MASH and atherosclerosis models suggest shared therapeutic opportunities, but do not establish that liver-derived LDs transmit inflammation to distant organs. pmc.ncbi.nlm.nih.gov Understanding whether these mechanisms operate locally or contribute to systemic disease remains an important translational question. Ultimately, therapeutic success will depend on limiting harmful inflammatory signaling while retaining the macrophage functions needed to clear injured tissue.
